# An unusual UMP C-5 methylase in nucleoside antibiotic polyoxin biosynthesis

**DOI:** 10.1007/s13238-016-0289-y

**Published:** 2016-07-14

**Authors:** Wenqing Chen, Yan Li, Jie Li, Lian Wu, Yan Li, Renxiao Wang, Zixin Deng, Jiahai Zhou

**Affiliations:** 1 Key Laboratory of Combinatorial Biosynthesis and Drug Discovery, Ministry of Education, and School of Pharmaceutical Sciences, Wuhan University, Wuhan, 430071 China; 2 State Key Laboratory of Bioorganic and Natural Products Chemistry, Shanghai Institute of Organic Chemistry, Chinese Academy of Sciences, Shanghai, 200032 China; 3 State Key Laboratory of Microbial Metabolism, and School of Life Sciences & Biotechnology, Shanghai Jiao Tong University, Shanghai, 200030 China

**Keywords:** polyoxin, nucleoside antibiotics, biosynthesis, UMP C5-methylase, thymidylate synthase

## Abstract

Polyoxin is a group of structurally-related peptidyl nucleoside antibiotics bearing C-5 modifications on the nucleoside skeleton. Although the structural diversity and bioactivity preference of polyoxin are, to some extent, affected by such modifications, the biosynthetic logic for their occurence remains obscure. Here we report the identification of PolB in polyoxin pathway as an unusual UMP C-5 methylase with thymidylate synthase activity which is responsible for the C-5 methylation of the nucleoside skeleton. To probe its molecular mechanism, we determined the crystal structures of PolB alone and in complexes with 5-Br UMP and 5-Br dUMP at 2.15 Å, 1.76 Å and 2.28 Å resolutions, respectively. Loop 1 (residues 117–131), Loop 2 (residues 192–201) and the substrate recognition peptide (residues 94–102) of PolB exhibit considerable conformational flexibility and adopt distinct structures upon binding to different substrate analogs. Consistent with the structural findings, a PolB homolog that harbors an identical function from *Streptomyces viridochromogenes* DSM 40736 was identified. The discovery of UMP C5-methylase opens the way to rational pathway engineering for polyoxin component optimization, and will also enrich the toolbox for natural nucleotide chemistry.

**Electronic supplementary material:**

The online version of this article (doi:10.1007/s13238-016-0289-y) contains supplementary material, which is available to authorized users.

## **INTRODUCTION**

Nucleoside natural products are a large family of microbial secondary metabolites with diverse bioactivities and unusual structural features (Isono, 1988; Niu and Tan, 2015; Chen et al., 2016). They have played distinguished roles in the treatment of the infections for mammalians and plants (Isono, 1988). Normally, the biosynthesis of nucleoside antibiotics follows a distinct logic via sequential modifications of the simple building blocks including nucleoside and nucleotide from primary metabolisms (Isono, 1988). Polyoxin, a group of structurally-related peptidyl nucleoside antibiotics, is produced by *Sreptomyces cacaoi* var. *asoensis* (*S. cacaoi* hereafter) and *Streptomyces aureochromogenes* (Chen et al., 2009). As the chemical structure of polyoxin mimics UDP-N-acetyl glucosamine, a building block for fungal chitin biosynthesis, it functions as a potent chitin synthetase inhibitor by targeting fungal cell wall biosynthesis (Endo and Misato, 1969; Endo et al., 1970). Polyoxin has therefore been widely used as an agricultural fungicide to control phytopathogenic fungi due to its distinctive action mode (Chen et al., 2009).

**Figure 1:**
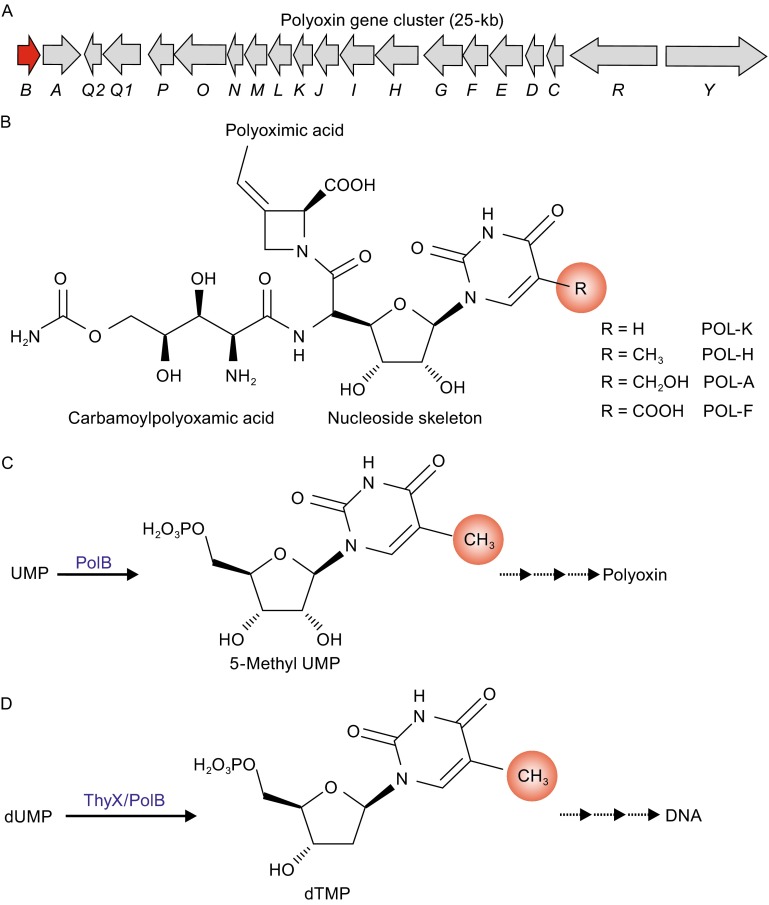
**Gene cluster and structure of polyoxin as well as the dual functions of PolB**. (A) The genetic organization of the polyoxin gene cluster. (B) The difference of polyoxin A, F, H and K is the C5 modification in nucleoside skeleton. (C) PolB catalyzes the UMP methylation in polyoxin biosynthesis. (D) ThyX and PolB could catalyze the dTMP biosynthesis. POL: Polyoxin

Polyoxin is composed of three moieties involving a nucleoside skeleton and two non-proteinogenic amino acids, carbamoylpolyoxamic acid and polyoximic acid (Fig. 1B) (Chen et al., 2009). The C-5 modifications within the nucleoside skeleton confer not only structural diversity but also possess a bioactivity preference for polyoxin (Isono et al., 1967; Isono and Suzuki, 1968; Isono et al., 1975; Zhai et al., 2012). Previous labeling studies indicated that the C-5 methylation originated from C-3 of serine and is catalyzed by a new enzyme independent of thymidylate synthase (Isono and Suhadolnik, 1976; Isono, 1988), however, the molecular mechanism for such modification remained elusive for decades.

We have previously identified the polyoxin biosynthetic gene cluster (Fig. 1A) from *S*. *cacaoi*, and tentatively proposed a pathway for the C5-methylation on the nucleoside skeleton (Chen et al., 2009). By analyzing the polyoxin biosynthetic gene cluster, we found that PolB, a thymidylate synthase (ThyX) homolog (Myllykallio et al., 2002; Graziani et al., 2004), is likely to be responsible for catalyzing the C5-methylation (Fig. 1C) (Chen et al., 2009). To dissect the function of PolB, we carried out a series of biochemical and crystallographic analysis which confirm PolB is an unusual flavin-dependent UMP/dUMP methylase (Fig. 1C and 1D). We solved the crystal structures of PolB as well as its complex structures with two substrate analogues 5-Br UMP or 5-Br dUMP. We found that the structure of PolB shares high similarity with its homologs ThyX proteins. However, the sequence identity is only 38%. Two special Loops, Loop 1 (residues 117–131) and Loop 2 (residues 192–201) which are highly conserved in primary sequence with ThyXs but not structurally existed in them are identified in PolB structure. Additional mutagenesis studies further reveal that residues Tyr124, Tyr126 and Tyr99 on Loop 1, Loop 2 and substrate recognition peptide (residues 94–102) are crucial for the catalytic activity and substrate selectivity of PolB. These results suggest that Loop 1 and Loop 2 cooperatively play a vital role in catalysis of UMP and dUMP methylation. Moreover, the findings of PolB as the first UMP methylase will shed light on the occurrence of C-5 modification for polyoxin biosynthesis and enrich the toolbox for thymidylate synthases.

## **RESULTS**

### ***polB is*responsible for the C5-methylation in polyoxin biosynthesis**

**Figure 2:**
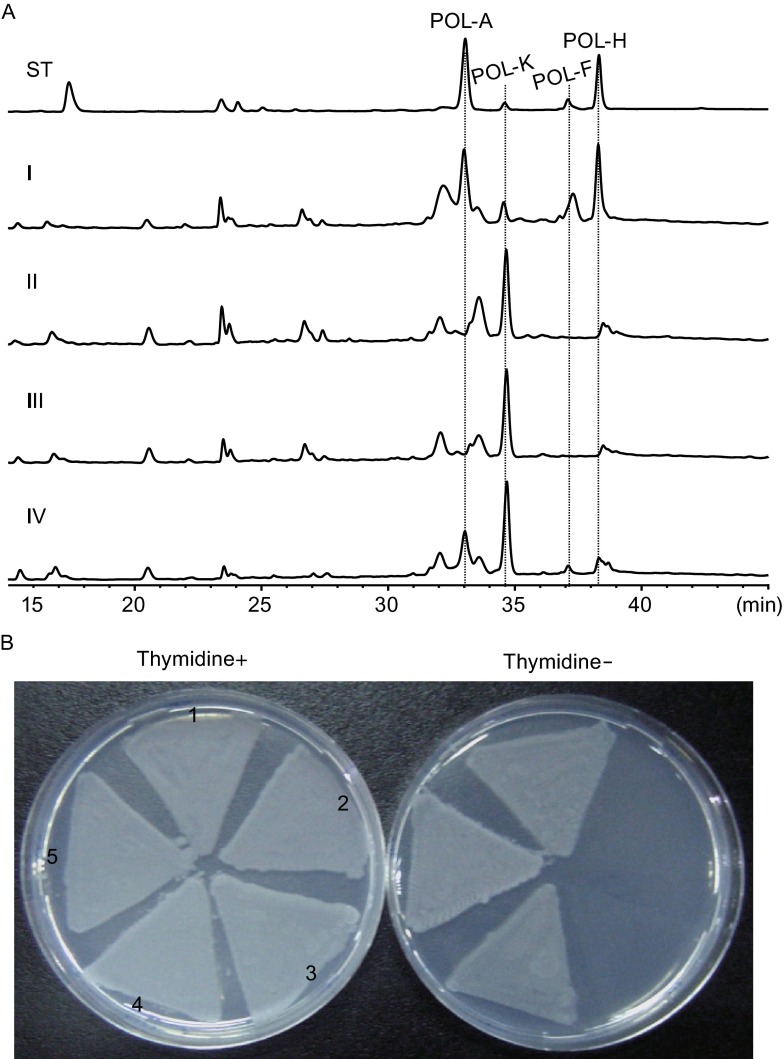
**Genetic characterization of the** ***polB*** **function**. (A) HPLC profiles of the metabolites produced by wild-type and mutant *S. cacaoi* strains. ST, polyoxin authentic standards; I, metabolites from wild-type *S. cacaoi*; II, metabolites from CY5; III, metabolites from CY5 containing pIB139 as negative control; IV, metabolites from CY5 complemented by *polB*. (B) *polB* is capable of restoring growth phenotype for the *thyX* mutant of *S. cacaoi*. 1: CY5; 2: CY6, the *thyX* and *polB* double mutant of *S. cacaoi*; 3: CY6/pIB139, CY6 containing pIB139 as negative control; 4: CY6/*polB*, CY6 complemented by *polB*; 5: CY6/*thyX*, CY6 complemented by *thyX*. The left plate contains thymidine, whereas which is absent in the right one

Bioinformatic analysis of polyoxin biosynthetic gene cluster revealed that *polB* is a potential candidate involved in performing C5 modification of the nucleoside skeleton (Fig. S1). To determine its involvement and the corresponding function, we first performed a gene knockout of *polB* in *S. cacaoi*. This was achieved via conjugation of a *polB* disruption vector pJTU2183 (Table S1) and a double-crossover replacement of the corresponding region in the chromosome of *S. cacaoi*. The sample of the resulting mutant CY5 was found to display higher bioactivity against the indicator strain *Trichosporon cutaneum* (Fig. S2A), implicating that the metabolites might be different. LC-MS analysis showed that CY5 was unable to produce polyoxin A (5-hydoxymethyl), polyoxin F (5-carboxyl) and polyoxin H (5-methyl), but instead accumulated polyoxin K (without C5 modification) (Figs. 2A and S2B–D). Complementation of the *polB* mutant CY5 restored the ability to produce polyoxin A, F and H, suggesting that *polB* is the target gene directly responsible for the C5-methylation of the nucleoside skeleton in polyoxin biosynthesis (Fig. 2A).

### **PolB harbors an alternative thymidylate synthase function**

A BLAST search for PolB homologs yielded sequences of several *Streptomyces* thymidylate synthases (ThyXs) (Fig. S1), suggesting that PolB may carry ThyX function. In bacteria, ThyXs catalyze the biosynthesis of dTMP by C5-methylation of dUMP, as dTMP is the key building block for DNA synthesis, deletion of *thyX* usually causes a lethal effect on cell growth in minimal medium. To evaluate the function of *polB*, we performed targeted gene knockout of *thyX* in the chromosome of *S. cacaoi*. Interestingly, CY3, the *thyX* mutant of *S. cacaoi*, survived and exhibited sporadic growth phenotype (Fig. S2E), implicating that PolB might complement the dTMP biosynthesis of ThyX. Indeed, when *thyX* and *polB* of *S. cacaoi* were both deleted, the resulting mutant CY6 could not grow at all in minimal medium (Fig. 2B).

### **Biochemical characterization of PolB as a FAD-dependent UMP/dUMP methylase**

**Figure 3:**
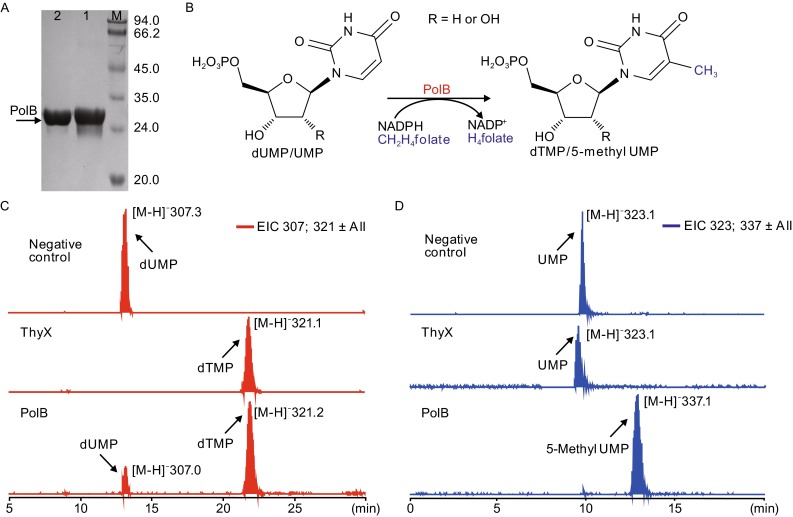
***In vitro*** **characterization of PolB as a UMP/dUMP methylase**. (A) SDS-PAGE analysis of PolB. M, molecular weight marker; lanes 1 and 2, purified His_6_-tagged PolB. LC-MS analysis of the reaction products of dUMP by ThyX or PolB. (B) Scheme of PolB catalyzed UMP/dUMP methylation reactions. (C) LC-MS analysis of the reaction products of ThyX or PolB using dUMP as substrate. Negative control, no PolB or ThyX was added into the reaction mixture. (D) LC-MS analysis of the reaction products of ThyX or PolB using UUMP as substrate. Negative control, no PolB or ThyX was added into the reaction mixture

To elucidate its biochemical role, we expressed *S. cacaoi* PolB as a N-terminal His_6_-tagged protein in *E. coli* BL21(DE3)/pLysE (Fig. 3A) and test its activity *in vitro*. The purified recombinant protein displayed bright yellow color, a characteristic of flavoprotein (Fig. S3A and S3B). By incubating PolB with dUMP, NADPH and CH_2_H_4_folate *in vitro*, we observed the target product of dTMP using LC-MS analysis (Figs. 3B, 3C and S3C–E). Further investigation revealed that PolB possessed UMP C5-methylase activity in the presence of NADPH and CH_2_H_4_folate (Figs. 3B, 3D, S3C, S3F and S3G). In comparison to PolB, recombinant *S. cacaoi* ThyX could catalyze the methylation of dUMP but not UMP when incubating with NADPH and CH_2_H_4_folate (Fig. 3C and 3D). These results demonstrated PolB as an unusual FAD-dependent UMP C5-methylase with thymidylate synthase activity.

Next, we measured the kinetic parameters of PolB to substrates UMP and dUMP (Fig. S3H). Although dUMP exhibits higher affinity (*K*_*m*_ = 12.96 ± 0.89 μmol/L) than UMP (*K*_*m*_ = 19.48 ± 4.15 μmol/L), the *k*_*cat*_ of UMP (*k*_*cat*_ = 3.09 ± 0.17 min^−1^) is 78% higher than that of dUMP (*k*_*cat*_ = 1.74 ± 0.05 min^−1^).

### **Structural comparison of PolB and ThyX**

**Figure 4:**
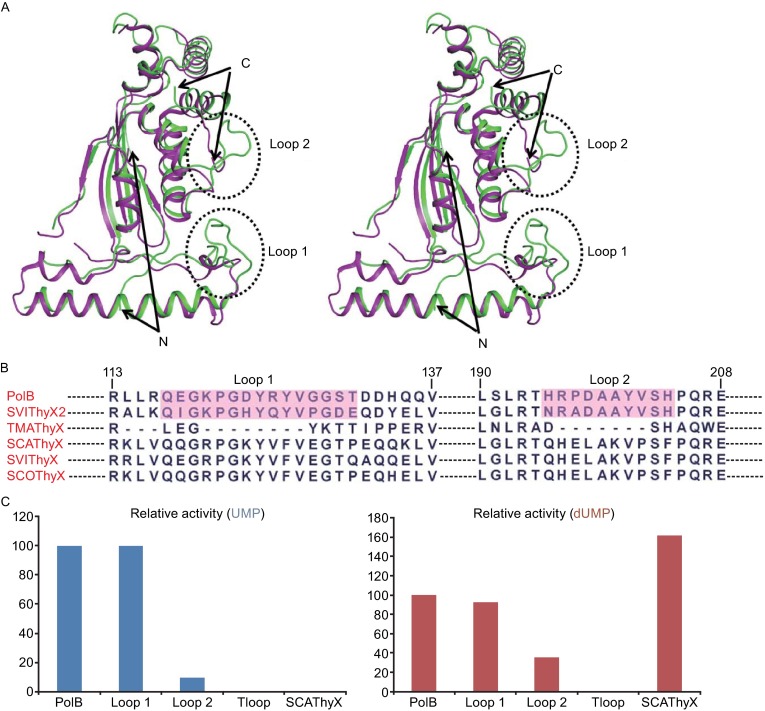
**Comparison of PolB and ThyX proteins**. (A) Superposition of PolB and TMAThyX (PDBID: 1O2A). PolB is shown in green, and TMAThyX is shown in purple. The N-and C-terminus are indicated. (B) Alignment of Loop 1 and Loop 2 between PolB and homologous ThyXs from *Streptomyces*. The residues in Loop 1 and Loop 2 of PolB and SVIThyX2 are boxed in magenta. (C) Comparison of the catalytic activities of ThyX, PolB and PolB variants for UMP (left panel) and dUMP (right panel). The relative activity was calculated on the basis of 3 repeats, and the error was all under control of ±5%

To investigate the molecular mechanism of PolB, we crystallized the protein and determined its crystal structure (abbreviated as apo-PolB in this work) using molecular replacement (McCoy et al., 2007) and refined at resolution of 2.15 Å (Table S3). Apo-PolB was crystallized in the *P*2_1_ space group with two tetramers in an asymmetric unit. Although there is only 38% sequence identity between PolB and ThyX from *Thermotoga maritima* (abbreviated as TMAThyX, PDB code: 1O2A) (Mathews et al., 2003), structure comparison revealed that the homotetramer of apo-PolB strongly resembles ThyX with an r.m.s.d less than 0.871 Å for 969 aligned Cα atoms. Especially, for the cofactor FAD binding pocket, little conformational change was observed between the two proteins (Fig. S4A). Nevertheless, a notable difference was observed in Loop 1 (residues 117–131) and Loop 2 (residues 192–201), which was not detected in TMAThyX, but presented high sequence conservation in other ThyXs (Fig. 4A and 4B). To probe the functional role of Loop 1 and Loop 2 in catalysis, we first generated PolB mutants by displacing the residues of these regions with the counterparts of *S. cacaoi* ThyX protein. The activity assay indicated that replacement of Loop 1 did not affect the enzymatic activity of PolB, while replacement of Loop 2 decreased the UMP methylation activity to 10% and the dUMP methylation activity to about 30%. When Loop 1 and Loop 2 were both replaced by the counterparts of *S. cacaoi* ThyX protein, the mutant PolB lost the methylation activity for both UMP and dUMP (Fig. 4C). This suggested that Loop 1 and Loop 2 are involved in regulating the catalytic activity of this methylase.

Other major differences between PolB and TMAThyX were in the N-terminus and the C-terminus (Figs. 4A and S1). In the PolB structure, the N-terminus adopts a flexible conformation while the C-terminus within the interior structure protrudes outside; in the TMAThyX structure (Mathews et al., 2003), the N-terminus forms a pair of anti-parallel β-sheets while the C-terminus extends outside.

### **Comparison of UMP and dUMP binding in the active site of PolB**

**Figure 5:**
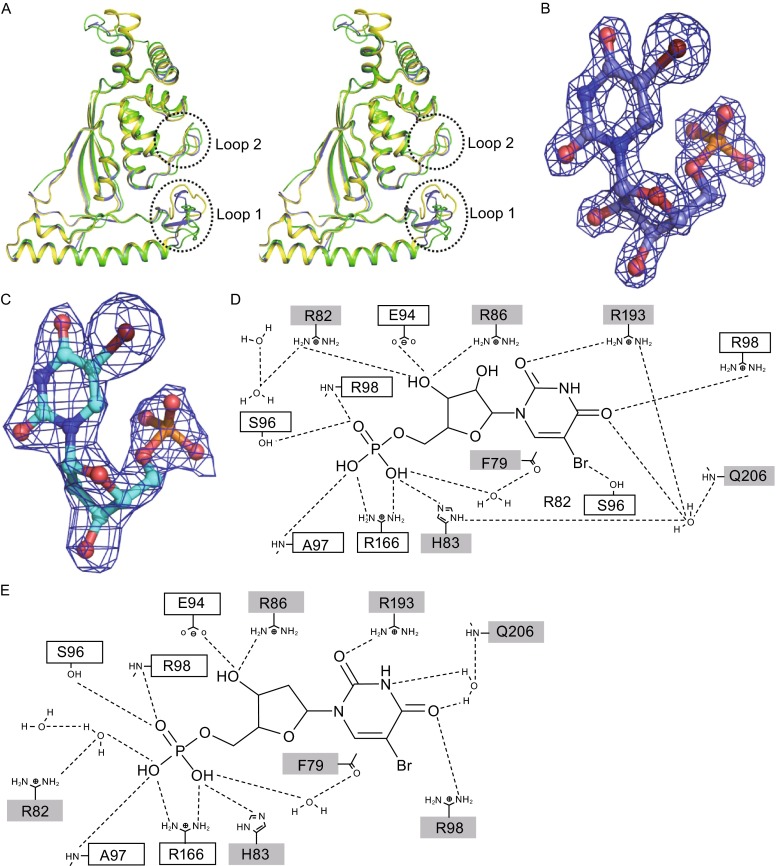
**Comparison of two structures of PolB complex**. (A) Superposition of structures of PolB (green), PolB/5-Br UMP (blue) and PolB/5-Br UMP (yellow). The N-and C-terminus are indicated. (B) and (C) Electron density for 5-Br UMP and 5-Br dUMP respectively. The 2*F*_o_-*F*_c_ map is contoured at 2.0 *σ*. (D and E) Schematic representations of the interactions between the active site residues of PolB and (D) 5-Br dUMP or (E) 5-Br UMP. The hydrogen bonds were labeled as dashed lines. Residues in the white and grey box are from two separate PolB monomers

To obtain the precise mechanism of PolB in UMP/dUMP methylation, we solved the structures in complex with substrate analogs 5-Br dUMP and 5-Br UMP at individual resolution of 2.28 Å and 1.76 Å (Table S3). Because the C5-position is substituted by the Br atom, these two structures should mimic the state of substrate binding. Both structures can be superimposed with the apo-PolB structure within rmsd of 1.26 Å over all the Cα atoms (Figs. 5A and S4B). In the tetrameric structure of PolB/5-Br dUMP, the thymine ring of the substrate 5-Br dUMP poses strong π-π interaction with the isoalloxazine moiety of FAD, and its phosphate group forms hydrogen bonds or salt bridges with the side chains of Phe79, Arg82, His83 from one monomer and Ser96’, Ala97’, Arg98’, Arg166’ from another neighboring monomer. Besides these interactions, the ribose O3’ is hydrogen bond to Glu94’ and Arg86; the pyrimidine O2 makes hydrogen bond to Arg193; the pyrimidine O4 has hydrogen bond to Arg98’ and water-mediated hydrogen-bond to Arg193 and Gln206 (Fig. 5D). The PolB/5-Br UMP complex structure is nearly identical in substrate binding to the PolB/5-Br dUMP complex structure except for an additional water-mediated hydrogen bond between ribose O3’ and Arg82 (Fig. 5E). Interestingly, the characteristic ribose O2’ in 5-Br UMP does not contact with any residues of PolB or water molecules. Therefore, little difference was observed in the active pocket of the structures of the two complexes.

**Figure 6:**
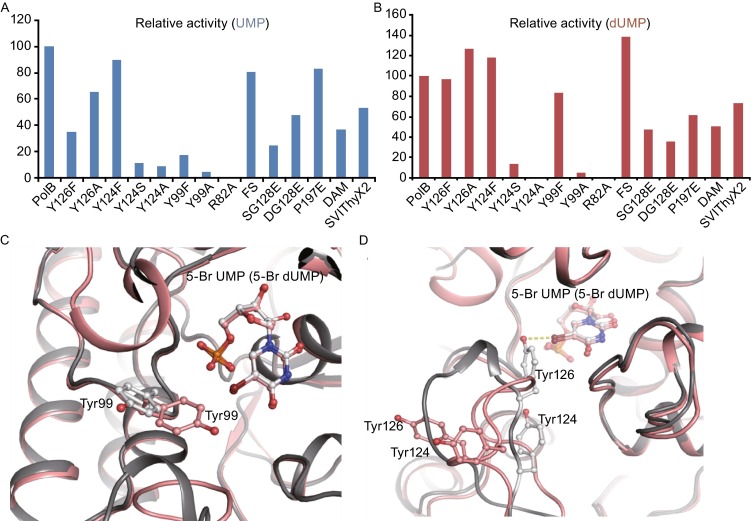
**Analysis of the roles of Loop 1 and Loop 2 in PolB catalysis**. (A) Comparison of the catalytic activities of PolB and its variants for UMP. (B) Comparison of the catalytic activities of PolB and its variants for dUMP. The relative activity was calculated on the basis of 3 repeats, and the error was all under control of ±5%. (C) Conformational change of Try99 in PolB-5-Br dUMP (salmon) and PolB-5-Br UMP (gray). (D) Conformation change of key residues Tyr124 and Tyr126 in Loop 1 around access while bound with 5-Br UMP (salmon) or 5-Br dUMP (gray)

Although the two PolB complex structures displayed almost identical catalytic mechanism, we perceived that Loop 1 and Loop 2 exhibit considerable conformational flexibility and are structurally distinct in two different complexes (Fig. 5A). Loop 1 undergoes dramatic conformational changes upon binding of either 5-Br UMP or 5-Br dUMP. This region becomes structurally ordered to form three short tandem β-sheets when the substrate analog 5-Br UMP binds to the active sites of PolB. Loop 2 undergoes obvious shift and adopts a stable conformation when either 5-Br UMP or 5-Br dUMP binds to PolB. We next screened a serial of site-directed mutants in Loop 1 and Loop 2 and measured their catalytic activities towards UMP and dUMP (Figs. 6A, 6B and S5A). We found that the conserved Tyr124 in Loop 1 was essential for catalysis while Tyr126 was necessary for substrate specificity. Mutation of Tyr124 to Phe did not affect the activity of PolB for either UMP or dUMP; however, replacement of this residue by Ala or Ser led to more than 90% activity loss for each substrate, indicating that the aromatic ring of Tyr124 is essential for catalysis (Fig. 6C and 6D). Tyr126 is only found in PolB while the corresponding residue in ThyX proteins of *Streptomyces* is phenylalanine (Fig. 4B). The Y126F mutant of PolB retained its full activity for dUMP methylation but lost over 60% of activity for catalyzing UMP, suggesting that this residue might be important for UMP methylation.

The third region varied between the structures of apo-PolB and two complexes was located in residues 94–102, whose counterpart in ThyX proteins was identified as the substrate recognition peptide (SRP). In apo-PolB, the electron densities of SRP were weak or difficult to observe. However, upon substrate binding, Glu94, Ser96, Ala97 and Arg98 in SRP are able to form hydrogen bonds to the substrate, which renders SRP ordered (Fig. S5B). We also discerned that the side chain of Arg98 in SRP forms hydrogen bond to Q206 in Loop 2. Notably, the side chain of Tyr99 in SRP adopts different rotamer structures in the two complex structures (Fig. 6C). In the PolB/5-Br dUMP structure, the hydroxyl group of the side chain points to the center of the active site and is close to the substrate. In contrast, for the structure of PolB/5-Br UMP, the hydroxyl group of the side chain points to the exterior of protein and is distant from the substrate. This suggested that the hydroxyl group of Tyr99 might be essential for substrate specificity. Indeed, the Tyr99F mutant kept 80% activity for dUMP but only 15% activity for UMP (Fig. 6A). As a control, the Tyr99A mutant almost abolished its activity for both UMP and dUMP.

### **Genome mining of PolB-like UMP methylase**

To firmly validate the role of Loop 1 and Loop 2 in dual-substrate specificities, we used them as probes for the mining of PolB-like proteins capable of producing 5-methyl UMP. BLAST search hits a putative thymidylate synthase (ID: ZP_07305627, designated as SVIThyX2) in *S. viridochromogenes* DSM 40736 with 71% identity to PolB (Fig. S1). The Loop 1 and Loop 2 regions in SVIThyX2 were different from those of ThyX proteins but highly conserved with the corresponding parts of PolB (Fig. 4B). The purified recombinant SCIThyX2 was incubated with NADPH, CH_2_H_4_folate and dUMP or UMP *in vitro*. LC-MS data showed that SVIThyX2 was able to catalyze the methylation for both UMP and dUMP (Figs. 6A, 6B and S6). These results demonstrated the PolB-like SVIThyX2 as the UMP methylase with thymidylate synthase activity, and further unambiguously confirmed the essential roles of Loop 1 and Loop 2 in UMP methylation.

## **DISCUSSION**

In this study, we demonstrated that PolB, combined with *in vivo* and *in vitro* assays, was able to catalyze the C5-methylation of both UMP and dUMP while the classic ThyXs only possess the dUMP methylase activity (Myllykallio et al., 2002). The crystal structures of PolB alone and in complex with the substrate analogs 5-Br UMP and 5-Br dUMP showed that the methylation mechanism of UMP and dUMP might be similar because they adopt the same binding pattern in the active site of PolB. The characteristic ribose O2’ in 5-Br UMP does not contact with any residues of PolB or water molecules. The structures indicate that Arg82, His83, Arg86, Glu94, Ser96, Arg98, Arg166, Arg193 and Gln206 play essential roles in PolB catalysis (Fig. 5D and 5E). Although UMP is the naturally preferred substrate of PolB, the kinetic studies and competitive binding experiments showed that dUMP rather than UMP exhibits higher affinity to PolB (Fig. S7). This is consistent with Frank Maley’s early reports on chick embryo thymidylate synthase half a century ago (Maley, 1960; Lorenson et al., 1967). The affinity difference between UMP and dUMP could be explained by steered molecular dynamics (SMD) simulation that dUMP rather than UMP need more external energy to dissociate from the protein (Fig. S8).

Analysis of the crystal structures of PolB indicates that three regions including Loop 1, Loop 2 and the substrate recognition peptide are crucial for the methylation of UMP/dUMP. They exhibit considerable conformational flexibility and became ordered to form a “closed” conformation by interacting with the substrate. Mutational studies uncovered that the phenyl group of Tyr99 in the substrate recognition peptide and Tyr124 in Loop 1 are essential for catalysis, consistent with the structural information that the benzene groups of Tyr124 and Tyr99 likely made π-π stacking interaction with the uracil ring of the substrate. Further mutational studies also demonstrated that the hydroxyl groups of Tyr99 in the substrate recognition peptide and Tyr126 in Loop 1 were important for substrate specificity. This is in full agreement with the SMD simulation results that showed the Y126F mutant decreases the success rate of UMP dissociation trajectories by 50% and increased the success rate of dUMP dissociation trajectories by 100% (Fig. S8). We proposed that Loop 1, Loop 2 and the substrate recognition peptide constituted the gate-keeper for substrate entrance and cooperatively regulated the catalysis of PolB on UMP/dUMP methylation. Our hypothesis that Loop 1 and Loop 2 regulated the substrate specificity of PolB was further validated by identification of SVIThyX2 as the second thymidylate synthase with UMP methylase activity.

The findings of PolB as a unique UMP methylase elucidated the origin of nucleoside skeleton C5-modification in polyoxin biosynthesis. Based on this work, we proposed that four different groups of polyoxins could be synthesized starting from UMP and its derivatives (5-methyl UMP, 5-hydroxymethyl UMP and 5-carboxyl UMP) via a potential pyrimidine salvage pathway. This hypothesis is supported by our previous report that when the biosynthetic gene cluster of polyoxin was heterologously expressed in *S. lividians* TK24, only the polyoxin H components were detected (Zhao et al., 2010). Co-expression of the polyoxin gene cluster of with *sav_4805* (encoding a thymine-7-hydroxylase homologous protein) from *S. avermitilis* (Omura et al., 2001) lead to the production of polyoxin A in *S. lividians* TK24. This indicated that genes responsible for the reaction from 5-methyl UMP to 5-hydroxymethyl UMP and 5-carboxyl UMP are not all adjacent to the polyoxin gene cluster. The *S. cacaoi* cells may also evolve or hijack a decarboxylase to convert 5-carboxyl UMP to normal UMP and complete the UMP salvage pathway. Further investigation of the component diversity of polyoxins will require all related genes in the metabolic pathway for 5-methyl UMP to be cloned.

In summary, we have reported the identification and structural basis of an unprecedented C5 methylase that employs FAD-dependent reductive mechanism for the methylation of UMP/dUMP. We also revealed that Loop 1, Loop 2 and substrate recognition peptide of the protein collectively constitute a gate-keeper for substrate selective-entrance and preferred-catalysis. The present data will provide insights for ThyXs evolution and enrich the chemical diversity of natural nucleotides.

## **MATERIALS AND METHODS**

### **Materials, methods and procedures**

All chemicals were from Sigma-Aldrich (IL, USA) unless otherwise indicated. 5-Br UMP, 5-Br dUMP and 5-methyl UMP were purchased from Hongene Biotechnology Ltd. (Shanghai, China). CH_2_H_4_folate was a gift from Merck. Materials and primers were individually listed in Table S1 and Table S2, and general methods and procedures were described by Kieser et al. (2000) and Sambrook et al. (1989).

### **Expression and purification of PolB and SVIThyX2**

All constructs and point mutations were generated using a standard PCR-based cloning strategy and verified through DNA sequencing. The recombinant PolB from *S. cacaoi* and SVIThyX2 from *S. viridochromogens* DSM40736 (Blodgett et al., 2005) were overexpressed at 30°C in *E. coli* BL21(DE3) as N-terminally His_6_-tagged proteins. The soluble fraction of the cell lysate was first purified using nickel affinity column (GE Healthcare) and further purified by gel-filtration chromatography (Superdex 75, HiLoad 16/60, GE Healthcare).

### **Activity assay**

All tests were performed in triplicates in 2 mL centrifuge tubes. A typical methylase activity assay (200 µL) contained 2.0 mmol/L NADPH, 0.2 mmol/L CH_2_H_4_folate, 0.2 mmol/L UMP (or dUMP), 50 mmol/L Tris-HCl (pH 8.0), and 10 μg of protein (PolB, ThyX or SVIThyX2). The reaction was terminated by adding TCA with final concentration 10% (*v*/*v*) and further analyzed by LC-MS using a ZORBA SB-C18 Column (5.0 μm, 4.6 × 250 mm, Agilent). The LC conditions were as follows. The elution buffer for LC was 10% methanol (*v*/*v*) contained 0.1% aqueous trifluoroacetic acid (*v*/*v*). The flow rate was 0.3 mL/min and the eluted fraction was monitored at 260 nm with a DAD detector. The parameters for MS analysis are 10 l/mL of drying gas flow, 30 psi of nebulizer pressure, and 325°C of drying gas temperature.

### **Crystallization and data collection**

All crystallization experiments were performed at 20°C using the sitting-drop vapor-diffusion method. Protein samples (12.5 mg/mL, 1 µL) stored in 25 mmol/L Tris-HCl, pH 8.0, 150 mmol/L NaCl and 5 mmol/L β-mercaptoethanol were mixed with well solution (1 µL) and equilibrated against the well solution (75 µL) in 96-well plates (HR3-271, Hampton Research). The crystal of apo-PolB was grown under the condition of 17% (*w*/*v*) PEG4000, 0.2 mol/L Li_2_SO_4_ and 0.1 mol/L Tris-HCl, pH 8.5. Crystals of the PolB/5-BrdUMP complex were obtained by mixing PolB (11.4 mg/mL) with 4 mmol/L of 5-Br dUMP and growing under the conditions of 1.4 mol/L sodium acetate and 0.1 mol/L sodium cacodylate, pH 6.5. Crystals of the PolB/5-Br UMP complex were obtained by mixing PolB (11.4 mg/mL) with 4 mmol/L of 5-Br UMP and growing under the conditions of 10% (*w*/*v*) PEG4000, 5% isopropanol and 0.1 mol/L HEPES, pH 7.5. Prior to data collection, all crystals were flash-cooled in liquid nitrogen using Paratone-N (HR2-463, Hampton Research) as cryo-protectants. Diffraction data were collected on a Mar225 detector at 100 K on the beamline BL17U1 at Shanghai Synchrotron Radiation Facility (Shanghai, China). The data sets were integrated and scaled with HKL2000.

### **Structure determination and refinement**

The structure of the PolB/5-Br dUMP complex was solved by molecular replacement using Phaser and the structure of TMAThyX (PDB code: 1O2A) as the search molecule. The structure of the PolB/5-Br UMP complex was solved by molecular replacement using Phaser and the PolB/5-Br dUMP complex as the search molecule. The structure of the PolB was solved by molecular replacement using Phaser and the PolB/5-Br UMP complex as the search molecule. Manual model building was performed with COOT (Emsley et al., 2010). Multiple rounds of refinement were carried out with Refmac5, CNS, and PHENIX. Noncrystallographic restraints were applied for one round of refinement. The overall quality of the final models was assessed by MolProbility and PROCHECK. Data collection and final refinement statistics are summarized in Table S3. All graphics were generated using PyMol.

### **Accession codes**

The crystal structures of PolB, the PolB/5-Br dUMP and the PolB/5-Br UMP have been deposited in the Protein Data Bank under accession number of 4P5C, 4P5B and 4P5A.

## **ACKNOWLEDGMENTS**

We are sincerely grateful to J. He, Q. Wang and S. Huang at SSRF BL17U1 beamline for data collection, and Prof. Zhihong Guo, Prof. Zong-Xiang Xia and Prof. Zhaohui Xu were appreciated for critical reading the manuscript. We’d also like to acknowledge Dr. Neil Price for assistance with MS data analysis, Prof. Shuangjun Lin for kinetic analysis of PolB and Xu-Dong Kong for help with figure preparation. This work was supported by grants from the National Basic Research Program (973 Program) (No. 2012CB721004 to W.C., No. 2011CB710800 to J.Z.), the National Grand Project for Medicine Innovation (2012ZX10002006 to J.Z.), the National Natural Science Foundation of Chin (Grant No. 31270100 to W.C.), Wuhan Youth Chenguang Program of Science and Technology (201507040401018 to W.C.).

## **ABBREVIATIONS**

SMD, steered molecular dynamics; SRP, substrate recognition peptide; ThyX, thymidylate synthase.

## **COMPLIANCE WITH ETHICS GUIDELINES**

The authors declare that they have no conflicts of interest pertaining to the contents of this article. This article does not contain any studies with human subjects performed by any of the authors.

## **AUTHOR CONTRIBUTIONS**

W.C., Y.L., J.Z. and Z.D. conceived the project. W.C. performed genetics experiments. W.C., Y.L. and S.L. performed biochemical experiments. Y.L. L.W. and J.L carried out crystallographic studies, Y.L. and R.X. calculated the molecular modeling data. W.C., Y.L., J.Z. and Z.D. analyzed data and wrote the manuscript.

## Electronic supplementary material

Below is the link to the electronic supplementary material.

Supplementary material 1 (PDF 2243 kb)
