# Supplementary material for: An unusual UMP C-5 methylase in nucleoside antibiotic polyoxin biosynthesis
Source: Protein Cell. 2016 Jul 14;7(9):673–83. doi: 10.1007/s13238-016-0289-y (PMC5003785; doi:10.1007/s13238-016-0289-y)
Supplement: Supplementary file 1 — Supplementary material 1 (PDF 2243 kb) [file 13238_2016_289_MOESM1_ESM.pdf]

---SUPPLEMENTAL DATA---

An Unusual UMP C-5 methylase in Nucleoside Antibiotic Polyoxin Biosynthesis

Wenqing Chen[1,§], Yan Li[2,§], Jie Li[2], Lian Wu[2], Yan Li[2], Renxiao Wang[2], Zixin Deng*[,1,3], Jiahai Zhou*[,2]

[1]Key Laboratory of Combinatorial Biosynthesis and Drug Discovery, Ministry of Education, and School of Pharmaceutical Sciences, Wuhan University, Wuhan 430071, China.

[2]State Key Laboratory of Bioorganic and Natural Products Chemistry, Shanghai Institute of Organic Chemistry, Chinese Academy of Sciences, Shanghai 200032, China.

[3]State Key Laboratory of Microbial Metabolism, and School of Life Sciences & Biotechnology, Shanghai Jiao Tong University, Shanghai 200030, China.

[§]These authors contributed equally to this work.

*To whom correspondence should be addressed: Jiahai Zhou, Tel:+86-21-54925377, Fax:+86-21-64166128, E-mail: jiahai@mail.sioc.ac.cn; Zixin Deng, Tel:+86-27-68756713, Fax:+86-27-68759850, E-mail: zxdeng@sjtu.edu.cn.

Supplemental Experimental Procedures

Construction and complementation of CY3 mutant For construction of CY3 mutant, left arm for in-frame deletion of *thyX* (Access no.JF268317) was amplified by primers cathyX-1F and cathyX-2R with KOD-plus (TOYOBO) polymerase. After that, the XbaI-BglII engineered PCR fragment was cloned into the corresponding sites of pOJ446 to generate pJTU2181, likewise, the BglII engineered PCR fragment amplified by the primers cathyX-3F and cathyX-4R was cloned into BglII-HpaI sites of pJTU2182 to result in the *S. cacaoi thyX* in-frame deletion vector, pJTU2182, which was subsequently transferred into *S. cacaoi* for in-frame deletion of *thyX* based on standard protocol(Kieser, 2000).

For complementation of CY3 mutant, *thyX* gene was amplified by the primers ca-thyx-EF and ca-thyx-ER, after digestion by EcoRI, this engineered fragment was cloned into EcoRI-SmaI site of pIJ2925 to give pJTU2174, and then the NdeI-EcoRI engineered fragment containing *thyX* was cloned into counterpart sites of pIB139 to produce pJTU2199 for complementation of CY3 mutant. Moreover, pIB139 was also conjugated into the CY3 mutant as negative control.

Construction and complementation of CY5 mutant For construction of CY5 mutant, a 1.8-kb PCR fragment amplified by polDf and polB2F primers, and then the XbaI-BglII engineered fragment was cloned into corresponding sites of pOJ446 to produce pJTU2831. After that, right arm amplified with primers polthyX1f and polB1R was cloned into HpaI-BglII sites of pJTU2831 to result in pJTU2832, which was further inserted into a BglII engineered fragment carrying *tsr* gene from pJTU2180 to generate the *polB* disruption vector, pJTU2833. Similarly,

CY5 was constructed according to standard methods of Kieser *et al* (Kieser, 2000).

For complementation of CY5 mutant, *polB* was firstly amplified by the primers po-thyx-eF and polBER with LA-Taq (Takara), then the PCR product was directly cloned into pMD18-T to give pJTU2175, then the NdeI-EcoRI engineered fragment containing *polB* was cloned into pIB139 to produce pJTU2872 for complementation of CY5 mutant.

Construction and complementation of CY6 mutant For construction of the mutant CY6, CY5 mutant was selected as start strain for further inactivation of *thyX*, accordingly, the BamHI engineered fragment harboring *neo* amplified by primers KanB-F and KanB-R was cloned into the corresponding site of pJTU2182 to result in pJTU2841, the *thyX* disruption vector. Subsequently, this disruption vector was conjugated into CY5 mutant for the targeted inactivation of *thyX* based on standard method. For complementation of the CY6 mutant, pJTU2199 and pJTU2872 was independently introduced into the mutant for investigation of respective phenotypes.

Kinetic analysis for PolB Substrate saturation kinetics were determined for UMP/dUMP (0, 5, 10, 20, 40, 80,160 and 320 μM), and the kinetic reaction for UMP/dUMP was carried out at fixed saturating concentrations of the other two substrates CH_2H_4folate (1.0 mM), and NADPH (2.0 mM). In all cases, 8 different concentrations of UMP/dUMP were used in each set of experimental assays with 2 μg PolB added in 100 μl reaction mix, which were then incubated at 30℃ for 20 min (UMP as substrate)/30 min (dUMP as substrate). After that, the PolB protein was precipitated by adding 10 μl 35 % trichloroacetic acid.

Site-directed mutagenesis. *polB* in pJTU2175 was site-mutated based on the method of overlapping PCR or according to the protocol of QuikChange® XL Site-Directed Mutagenesis Kit (Stratagene). All potential mutation constructs were confirmed by DNA sequencing, and then the variants of *polB* were cloned into pET28a for further functional validation. Loop1, "D_{123}YRYVGGSTDD_{133}" were changed to "K_{123}YVFVEGTPEQ_{133}"; loop2, "H_{195}RPDAAYVSH_{204}" were changed to "Q_{195}HELAKVPSF_{204}"; TLoop, Loop1 and Loop2 were both changed described as above; FS, F234N and S238A were indicated. DAN, DAN38-40 was mutated to QSL; DG128E, Y126 and G128 were simultaneously mutated to "Y126F&G128E".

Molecular dynamics (MD) simulations preparation The intact tetrameric structures of PolB protein complexed with 5-Br UMP and 5-Br dUMP were first constructed, which were used as the initial models for MD simulations. 5-Br UMP, 5-Br dUMP and the cofactor FAD were extracted from the crystal structures. The electrostatic potentials of each ligand were obtained through single-point energy calculation at the HF/6-31g (d) level by using the Gaussion03 program, and the atomic partial charges on the ligand were derived by the RESP method in the AMBER program (version 9). Atoms on PolB protein were assigned the template charges provided in AMBER force field, and all ionizable residues were set at their default protonation states at pH7.0. After that, the all-atom models for PolB/5-Br UMP and PolB/5-Br dUMP were generated using the tleap module in AMBER9. The resulted models were solvated with TIP3P water in a cubic periodic box with a margin of 10 Å along each dimension. An appropriate number of counter ions were added to neutralize the whole systems. Protocols for MD simulation were set as following: The protein and ligand were

constrained by a force constant of 10 kcal/mol·Å. Minimization of the system was performed until the root-mean-square of the Cartesian elements of the gradient is less than 0.01 kcal/mol·Å. Then, the system was further minimized with no constraints until the root-mean-square of the Cartesian elements of the gradient is less than 0.01 kcal/mol·Å. After the relaxation, a 120 ps simulation was used to gradually raise the temperature of the system from 0 to 300 K with no constraints. Another 30 ps was used for equilibrating at 300 K. A subsequent 3 ns production run was performed under a constant temperature of 300 K and a constant pressure of 1 atm. The time interval was set to 2 fs. The General AMBER Force Field (GAFF) was applied in simulation with the Particle Mesh Ewald (PME) method[9] for handling long-range electrostatic interactions and the SHAKE algorithm[10] for constraining all covalent bonds connecting hydrogen atoms. The non-bonded cutoff distance was set at 12 Å. No other constraint was applied to either the protein or the ligand during the entire MD simulation. All the MD simulations were performed on an Intel Xeon 5345-based Linux cluster.

Steered molecular dynamics (SMD) The final conformations derived from previous MD simulations were used as initial models for SMD simulations. 1 ns SMD simulations were performed under the NPT ensemble and Langevin dynamics, and external restraints were applied to pull the ligand out of the active site of PolB protein. Based on several preliminary trials, the pulling direction was defined along a vector formed between the initial position of 5-Br UMP/5-Br dUMP and the Cα atom of Leu298 (L548 in the model of PolB/5-Br dUMP). The constant-velocity SMD was used in the current simulations. The pulling velocity was set

as 0.03 Å/ps, and the restraint constant was set as 50 kcal/mol·Å^2. To avoid the translation and rotation, three Cα atoms of R45, Q122 and T205 were restrained by a harmonic potential with a force constant of 50 kcal/mol·Å^2. The corresponding residues are R757, Q834 and T917 in the model of PolB/5-Br dUMP. SMD simulations were repeatedly carried out with different random seeds for computing the force and work. To explain the different effects of residue mutations (Y99F and Y126F, residue number from original structure) for catalyzing substrates UMP and dUMP, SMD simulations for the mutated PolB complex with 5-Br UMP/5-Br dUMP were performed in parallel.

Table S1. Strains, plasmids and cosmids used in this study

Strain/ Plasmid/ Cosmid	Relevant characteristics*	Reference or source
***S. cacaoi* strains**		
WT	Wild-type of polyoxin producing strain	(Chen et al., 2009)
CY3	In-frame deletion mutant of *thyX*	This study
CY3/pIB139	CY3 mutant containing pIB139	This study
CY3/pJTU2199	CY3 mutant containing pJTU2199	This study
CY5	*polB* disruptant generated by insertion of *tsr*	This study
CY5/pIB139	CY5 mutant containing pIB139	This study
CY5/pJTU2872	CY5 mutant containing pJTU2872	This study
CY5/pJTU2803	CY5 mutant containing pJTU2803	This study
CY6	*polB* and *thyX* double mutant	This study
CY6/pIB139	CY6 mutant containing pIB139	This study
CY6/pJTU2872	CY6 mutant containing pJTU2872	This study
CY6/pJTU2199	CY6 mutant containing pJTU2199	This study
***E. coli* strains**		
DH10B	F^{-}*mcr*A Δ (*mrr-hsd*RMS-*mcr*BC) ϕ80d *lac*ZΔ M15 Δ *lac*X74 *deo*R*rec*A1*end*A1*ara*Δ139 D(*ara*, *leu*)1697 *gal*U*gal*Kλ^{-}*rps*L*nup*G	GIBCO BRL
ET12567/pUZ8002	*dam dcmhsd*S pUZ8002	(Kieser, 2000)
BL21(DE3)pLysE	F^{-} ,*ompT, hsdS*$_B$ *(*r_B^{-} m_B^{-}*)* ,*gal* ,*dcm* (DE3), pLysE (Cm^R)	Stratagene
Trichosporon cutaneum	Indicator fungi used for bioassay of polyoxin	CGMCC
**Plasmids**		
pIJ2925	*bla, lac*Z	(Kieser, 2000)
pBlueScriptII SK(+)	*bla, lac*Z, *ori*f1	Stratagene
pMD18-T	pUC18 derivative, T-vector	TaKaRa
pSET152	*aac(3)IV*, *lacZ*, *rep*^{pMB1*} attΦC31, *ori T*	(Kieser, 2000)
pOJ446	*aa (3)I V*, SCP2, *rep*^{pMB1*}, attΦC31, *ori* T	(Kieser, 2000)
pJTU1278	pHZ1358 derivative, *bla, lac*Z, *tsr,ori* T	(Kieser, 2000)
pHZ1070	pIJ2925 derivative carrying *aac(3)IV*	(Chen et al., 2009)
pET28a	Kan, *rep* ^{pMB1}, T7 promoter	Novagen
pJTU2174	pIJ2925 derivative containing structural gene of *S.*	This study

	cacaoi thyX	
pJTU2175	pMD18-T derivative carrying *polB* structural gene	This study
pJTU2176	pET28a derivative with insertion of NdeI-EcoRI engineered fragment containing *polB* from pJTU2175	This study
pJTU2177	pET28a derivative with insertion of NdeI-EcoRI engineered fragment containing *S. cacaoi thyX* from pJTU2174	This study
pJTU2180+	pJTU2925 derivative carrying BclI engineered fragment containing *tsr* gene from pJTU1278	This study
pJTU2181	pOJ446 derivative with insertion of XbaI-BglII engineered PCR fragment containing left arm for in frame deletion of *S. cacaoi thyX*	This study
pJTU2182	pJTU2181 derivative carrying a BglII engineered PCR fragment containing right arm for in frame deletion of *S. cacaoi thyX*	This study
pJTU2193	pJTU2182 derivative with insertion of a BglII fragment containing *tsr* from pJTU2180+	This study
pJTU2199	pIB139 derivative carrying a NdeI-EcoRI fragment containing *thyX (S. cacaoi)* from pJTU2174	This study
pJTU2803	pIB139 derivative carrying a NdeI-EcoRI fragment containing *polB* encoding 257-aa	This study
pJTU2826	pJTU2170 derivative carrying a NdeI-EcoRI fragment containing *S. cacaoi thyX* from pJTU2199	This study
pJTU2831	pOJ446 derivative carrying 1.8-kb XbaI-BglII engineered PCR fragment using as left arm for *polB* disruption	This study
pJTU2832	pJTU2831 derivative carrying 1.8-kb BglII engineered PCR fragment using as right arm for *polB* disruption	This study
pJTU2833	pJTU2832 derivative carrying a BglII engineered fragment containing tsr from pJTU2180+	This study
pJTU2841	pJTU2182 derivative carrying a BamHI engineered PCR fragment containing *neo*	This study
pJTU4870	SK+ derivative with the *SVIthyX2* (*S. viridochromogenes*) gene (ZP_07305627.1) cloned into EcoRI-SmaI site	This study
pET28a/SVIThyX2	pET28a derivative with insertion of the engineered EcoRI-SmaI fragment containing *SVIthyX2* (*S. viridochromogenes*) gene	This study
**Cosmids**		

4H4	Positive cosmid containing *thyX* gene of *S.cacaoi*	This study
13H3	Positive cosmid containing *thyX* gene of *S.cacaoi*	This study

*ori*T, origin of transfer of plasmid RK2; *tsr*, thiostrepton resistance gene; *aac(3)IV,* apramycin resistance gene; Cm^R, chloramphenicol resistance gene; *neo*, Neomycin resistance gene; Kan, kanamycin resistance gene; CGMCC, China General Microbiological Culture Collection Center; rep^{pMB1*}, mutated rep^{pMB1}.

Table S2. PCR primers used in this study

Primers	Sequence
PtsF	5'-GACGAGCTGAAGAAGGACCCG-3'
PtsR	5'-CCACT CCGCC TCCATCTTCTC-3'
ca-thyx-eF	5'-GGCATATGACCGACAGCCCCGCCGA-3'
ca-thyx-ER	5'-GGAATTCTTCAGGGGGCGACGCGGC-3'
po-thyx-eF	5'-GGCATATGGAGTCACCCCGGATCG-3'
polBER,	5'-GGAATTCTTCACGGGCTGACGCGGC-3'
cathyX-1F	5'-GCTCTAGAGCCGTCCTCACCATGAAGT-3'
cathyX-2R	5'-GAAGATCTCAGTTCGACGGTGACATC-3'
cathyX-3F	5'-GAAGATCTGAGCAGTCCTACCTCCAG-3'
cathyX-4R	5'--GAGGGTGTACGGGCGGA-3'
pothyXIFD-1F	5'-GGAATTCGACTTCACCCTCCGCT
polB1R	5'-GAAGATCTCCCGAGCCGCCTTGACGA-3'
polB2F	5'-GAAGATCTCGGTCACCCACGAGGCGTT-3'
podfF	5'-GCTCTAGA CTGTTCGTCTGGCTGGACC-3'
cathyXIFD-f	5'-GAAGACGCGACGGAAGGA-3'
cathyxIFD-R	5'-AATGCCGCAATACGGACAC-3'
polB-F2	5'-CCATATGACGGAGTTCCCCGC-3'
KanB-F	5'-CGGGATCCAGCTATTCCAGAAGTAGT-3'
KanB-R	5'-CGGGATCCTGGATGCCGACGGATTTG-3'
R82A-F	5' -CATGAGGTGGGCCACGGTGAA-3'
R82A-R	5' -CACCGTGGCCCACCTCATGC-3'
Y99A-F	5' -GAGGAGAGCGCCCGGGCCCGCGAGGTC-3'
Y99A-R	5' -GACCTCGCGGGCCCGGGCGCTCTCCTC -3'
Y99F-F	5' -GAGGAGAGCGCCCGGTTCCGCGAGGTC-3'
Y99F-R	5' -GACCTCGCGGAACCGGGCGCTCTCCTC-3'
Y124A-F	5'-AAGCCCGGCGACGCCCGGTA-3'
Y124A-R	5'-TACCGGGCGTCGCCGGGCTT-3'

Y124F-F	5'-AAGCCCGGCGACTTCCGGTA-3'
Y124F-R	5'-TACCGGAAGTCGCCGGGCTT-3'
Y124S-F	5'-AAGCCCGGCGACTCCCGGTA-3'
Y124S-R	5'-TACCGGGAGTCGCCGGGCTT-3'
Y126A-F	5'-GGC GAC TAC CGG TAC GTC GGC GGC AGC-3'
Y126A-R	5'-GGC GAC TAC CGG GCC GTC GGC GGC AGC-3'
Y126F-F	5'-CGGCGACTACCGGTTCGTCGGCGGCAGCA-3'
Y126F-R	5'-TGCTGCCGCCGACGAACCGGTAGTCGCCG-3'
P197E-F	5'-GCGCACCCACCGGGAGGACGCGGCCTACG-3'
P197E-R	5'-CGTAGGCCGCGTCCTCCCGGTGGGTGCGC-3'
G128E-F	5'-CTACCGGTACGTCGAAGGCAGCACCGAC-3'
G128E-R	5'-GTCGGTGCTGCCTTCGACGTACCGGTAG-3'
DG128E-F	5'-TACCGGGCCGTCGAAGGCAGCACCGAC-3'
DG128E-R	5'-GTCGGTGCTGCCTTCGACGGCCCGGTA-3'
DAN38-40QSL-F	5'-CACCGCCGGTGAACAGTCCCTCGACGAGCTGTACG-3'
DAN38-40QSL-R	5'-CGTACAGCTCGTCGAGGGACTGTTCACCGGCGGTG-3'
F234N S238A-R	5'-GGAAtTCACGGGGCGACGCGGCCGTTGGC-3'
Loop1-F	5'-TACGTCTTCGTCGAGGGCACGCCGG<br>AGCAGCACCAGCAGGTGGTCCGGT-3'
Loop1-R	5'-CTCCGGCGTGCCCTCGACGAAGAC<br>GTACTTGCCGGGCTTGCCCTCCTGG-3'
Loop2-F	5'-CAGCACGAACTGGCGAAGGTCCCG<br>TCCTTCCCCCAGCGGGAGATCGAGA-3'
Loop2-R	5'-GAAGGACGGGACCTTCGCCAGTTC<br>GTGCTGGGTGCGCAGGCTGAGGAA-3'
SVIThyX2-EF	5'-CCATATGACGATCAGCCCGGCCAT-3'
SVIThyX2-ER	5'-GGAATTCAGGGGCTGACCCGGCCGA-3'

Table S3. Data collection and refinement statistics

	apo-PolB	PolB/5-Br dUMP	PolB/5-Br UMP
**PDB ID**	4P5C	4P5B	4P5A
**Data collection statistics**			
Space group	$P2_1$	$C2$	$P2_1$
Wavelength (Å)	0.9796	0.9796	0.9796
Cell dimensions			
a,b,c (Å)	59.695, 166.123, 104.360	170.788, 59.502, 118.981	62.897, 94.160, 91.948
α,β,γ(°)	90.0, 101.7, 90.0	90.0, 120.3, 90.0	90.0, 98.3, 90.0
Resolution (Å)	50-2.15 (2.23-2.15)	50-2.28 (2.36-2.28)	50-1.76 (1.82-1.76)
Rmerge (%) [a]	7.9 (45.5)	11.5 (42.4)	8.7 (44.9)
Mean <I/σ(I)>	9.86 (2.05)	11.0 (4.52)	13.49 (5.96)
Refelctions (Measured/Unique)	574249/91297	136497/46721	775223/103888
Completeness (%)	84.5 (58.1)	98.0(98.3)	98.7(97.6)
Redundancy	6.3 (3.8)	2.9 (2.8)	7.5 (7.1)
**Refinement Statistics**			
R-factor (%)/free R-factor (%)[b]	20.1/25.6	16.4/21.5	15.3/19.2
Number of water molecular	160	402	733
Average B factors (Protein/water, $Å^2$)	61.88/55.33	31.31/33.60	18.6/26.74
R.m.s deviations			
Bond lengths (Å)	0.008	0.008	0.007
Bond angles (°)	1.161	1.237	1.181
Ramachandran (%)			
Most favored	93.78	96.48	98.05
Generously allowed	4.87	3.08	1.84
Disallowed	1.35	0.44	0.11

[a]$R_{merge} = \sum | I_i - \langle I \rangle | / \sum I_i$, where I_i is the intensity of an observation and $\langle I \rangle$ in the mean value for this reflection, and the summations are over all reflections. Values in parentheses are for the highest resolution shell.

[b]*R*-factor $= \sum_h | Fo(h) - Fc(h) | / \sum_h Fo(h)$, where *F*o and *F*c are the observed and calculated structure factor amplitudes, respectively. The free *R*-factor was calculated with 5% of the data excluded from refinement.

Supplemental Results

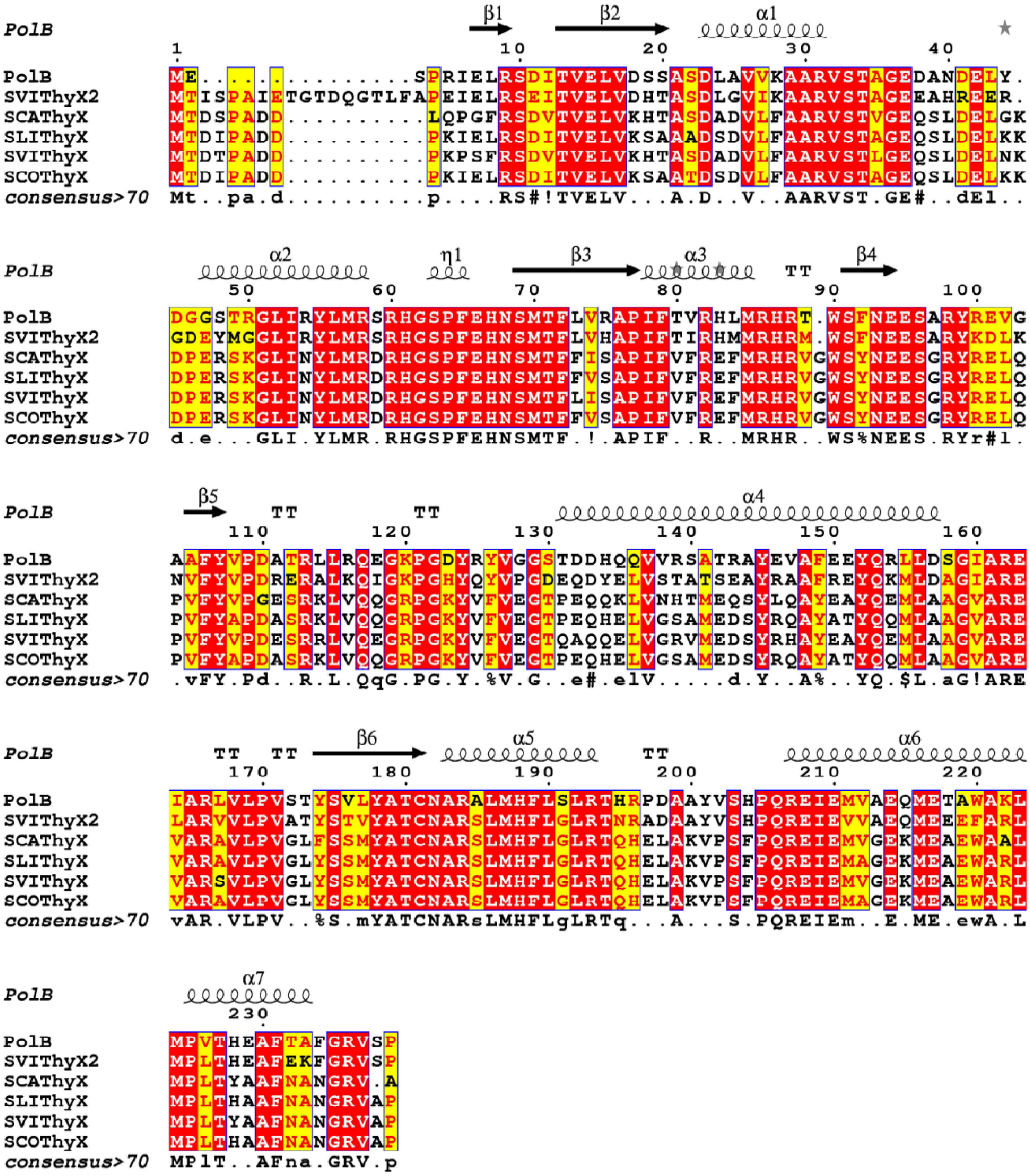

Figure S1. Sequence alignment of PolB and related ThyX proteins. Proteins include PolB, *S. cacaoi*; SVIThyX2 (SSQG_04514, accession no ZP_07305627.1), *Streptomyces viridochromogenes* DSM 40736; SCAThyX, *S. cacaoi*; SLIThyX, *Streptomyces lividans* TK24; SVIThyX, *Streptomyces viridochromogenes* DSM 40736; SCOThyX, *S. coelicolor* A3(2).The sequences were aligned with ClustalW and the results were merged with ESPript. The secondary structure of PolB from this work was shown on top of the alignment results.

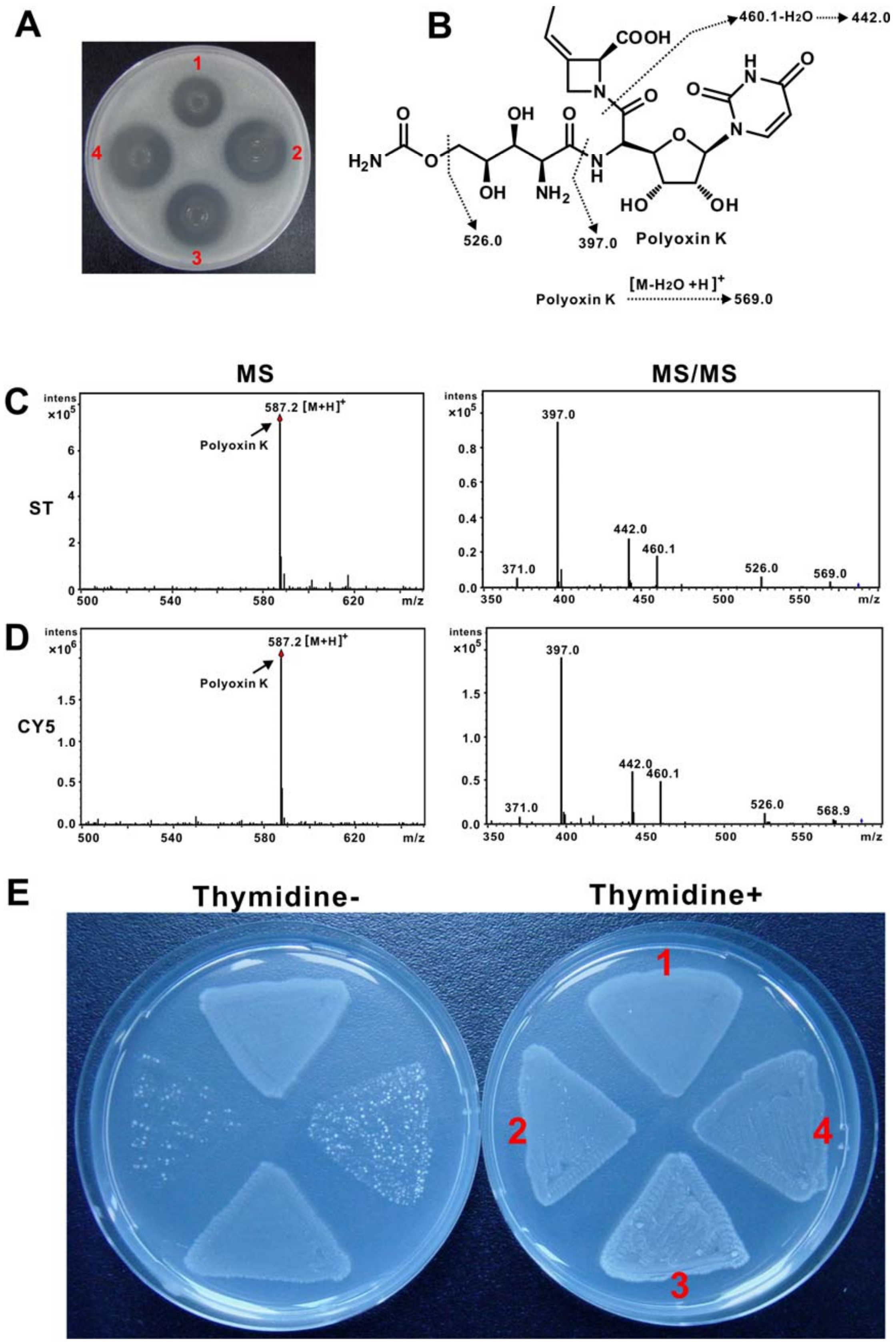

Figure S2. Analysis of the phenotypes of *S. cacaoi* CY5 and CY3 mutants. (A) Bioassay of the metabolites produced by *S. cacaoi* and its relevant recombinant strains. 1: metabolites from

wild-type S. cacaoi; 2: metabolites from the polB mutant CY5 of S. cacaoi; 3: metabolites from CY5 containing pIB139 as negative control; 4: metabolites from CY5 mutant complemented by polB. **(B)** Fragmentation pattern of polyoxin K component**.** **(C)** MS and MS/MS analysis of the polyoxin K authentic standard, which generates a characteristic $[M+H]^+$ ion at *m/z* 587.2 with principal fragmentation pattern at *m/z* 371.0, 397.0,442.0,460.1,526.0 and 569.0.**(D)** MS and MS/MS analysis of the metabolites produced by CY5 mutant, which generates a characteristic $[M+H]^+$ ion at *m/z* 587.2 with principal fragmentation pattern at *m/z* 371.0, 397.0, 442.0, 460.1, 526.0 and 568.9, fully corresponding to those of polyoxin K authentic standard. **(E)** Surface-grown experiment for CY3 mutant and its related strains on MM, 1, Wild type; 2, CY3; 3, CY3 containing pJTU2199 as *thyX* complemented strain; 4, CY3 containing pIB139 as negative control.

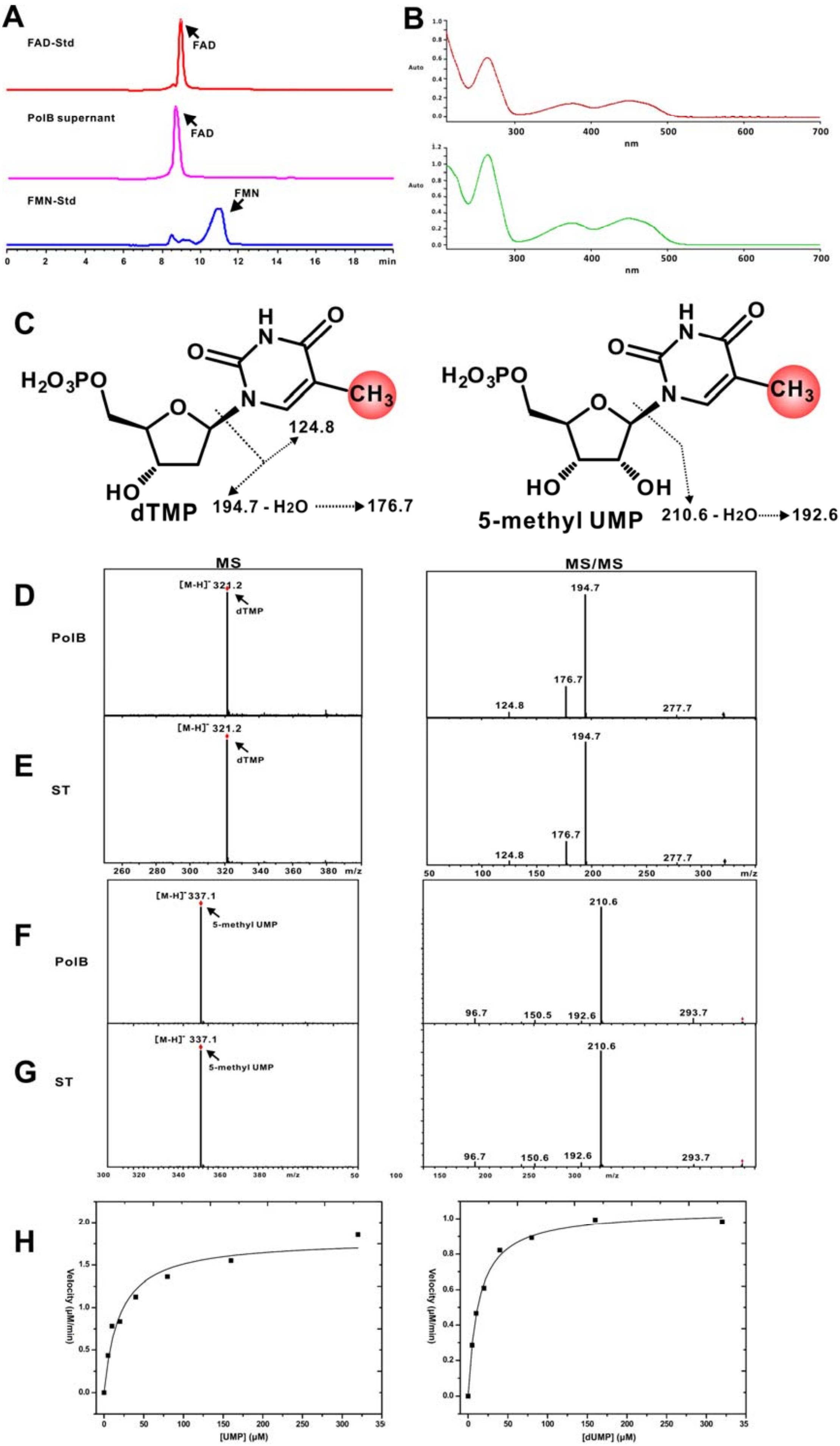
A
FAD-Std
FAD
PolB supernant
FAD
FMN-Std
FMN
min
B
Auto
nm
C
H_2O_3PO
HO
dTMP
124.8
194.7 - H2O
176.7
5-methyl UMP
OH
210.6 - H2O
192.6
MS
MS/MS
D
PolB
[M-H]⁻ 321.2
dTMP
194.7
176.7
124.8
277.7
E
ST
m/z
F
PolB
[M-H]⁻ 337.1
5-methyl UMP
210.6
96.7
150.5
192.6
293.7
G
ST
150.6
H
Velocity (μM/min)
[UMP] (μM)
[dUMP] (μM)

Figure S3.Biochemical characterization of PolB and its-reactions. (A) HPLC analysis of PolB-bound cofactor, FAD-Std, FAD authentic standard; PolB supernant, Supernant sample from PolB, which was inactivated by heating at 95 ℃ for 10min; FMN-Std, FMN authentic standard. FAD was detected at 266 nm. (**B**) Spectrometry analysis of PolB-bound cofactor. Top: the FAD authetntic standard, Bottom: Supernant sample from PolB, which was inactivated by heating at 95 ℃ for 10min; **(C)** Proposed fragmentation patterns for 5-methyl UMP and dTMP.**(D)** MS and MS/MS analysis of the PolB catalyzed product, which was capable of generating a target $[M\text{-}H]^-$ ion at *m/z* 337.1 with major fragmentation pattern at *m/z* 96.7, 150.5, 192.6, 210.6 and 293.7. **(E)** MS and MS/MS analysis of 5-methyl UMP authentic standard, which generated $[M\text{-}H]^-$ ion at *m/z* 337.1 with major fragment ion at m/z 96.7, 150.6, 192.6, 210.6 and 293.7. As this figure indicated, the MS and MS/MS pattern of PolB catalyzed product is fully consistent with that of the 5-methyl UMP authentic standard, undoubtedly demonstrating the identity of the PolB catalyzed product as 5-methyl UMP. **(F)** MS and MS/MS analysis of the PolB catalyzed product, which was capable of generating a target $[M\text{-}H]^-$ ion at *m/z* 337.1 with major fragmentation pattern at *m/z* 96.7, 150.5, 192.6, 210.6 and 293.7. **(G)** MS and MS/MS analysis of 5-methyl UMP authentic standard, which generated $[M\text{-}H]^-$ ion at *m/z* 337.1 with major fragment ion at m/z 96.7, 150.6, 192.6, 210.6 and 293.7. **(H)** Single-substrate kinetic analysis using variable UMP (0-320 μM) (Left) and dUMP (0-320 μM) (Right).

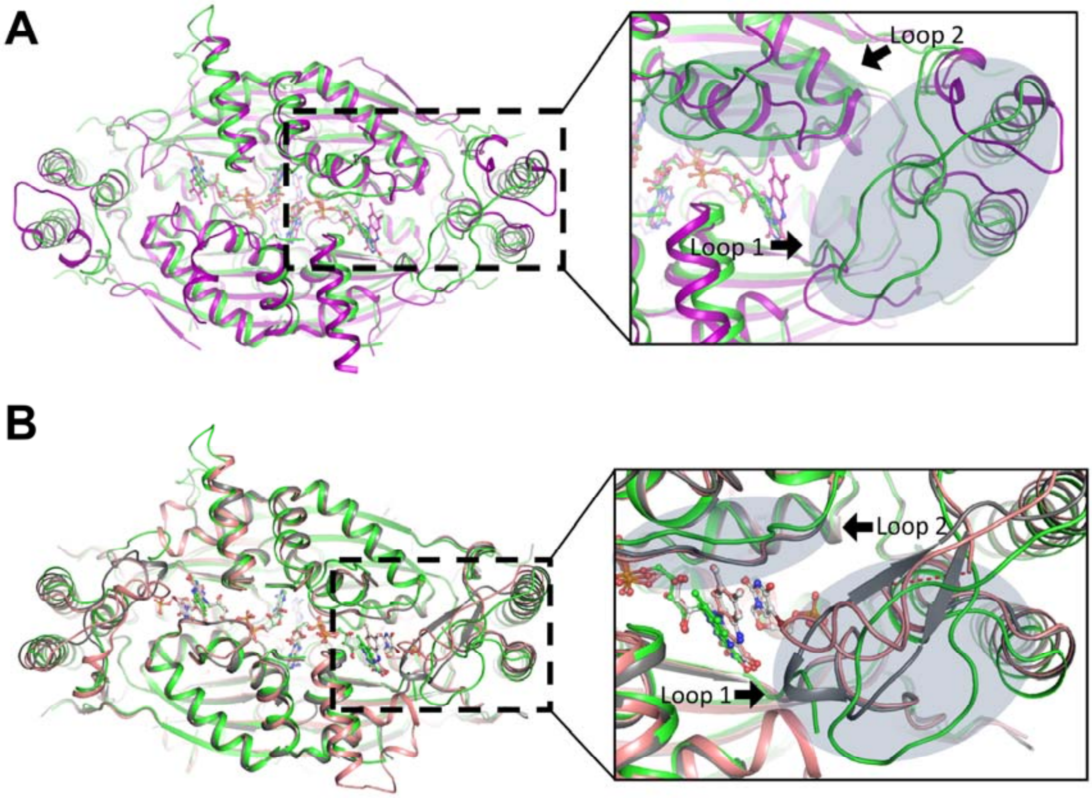

Figure S4. Structural analysis of PolB. (**A**) Superposition of the tetramer structure of PolB (green) and TMAThyX (purple). (**B**) Comparison of apo-PolB (green), PolB/5-Br dUMP (salmon) and 5-Br UMP (gray).

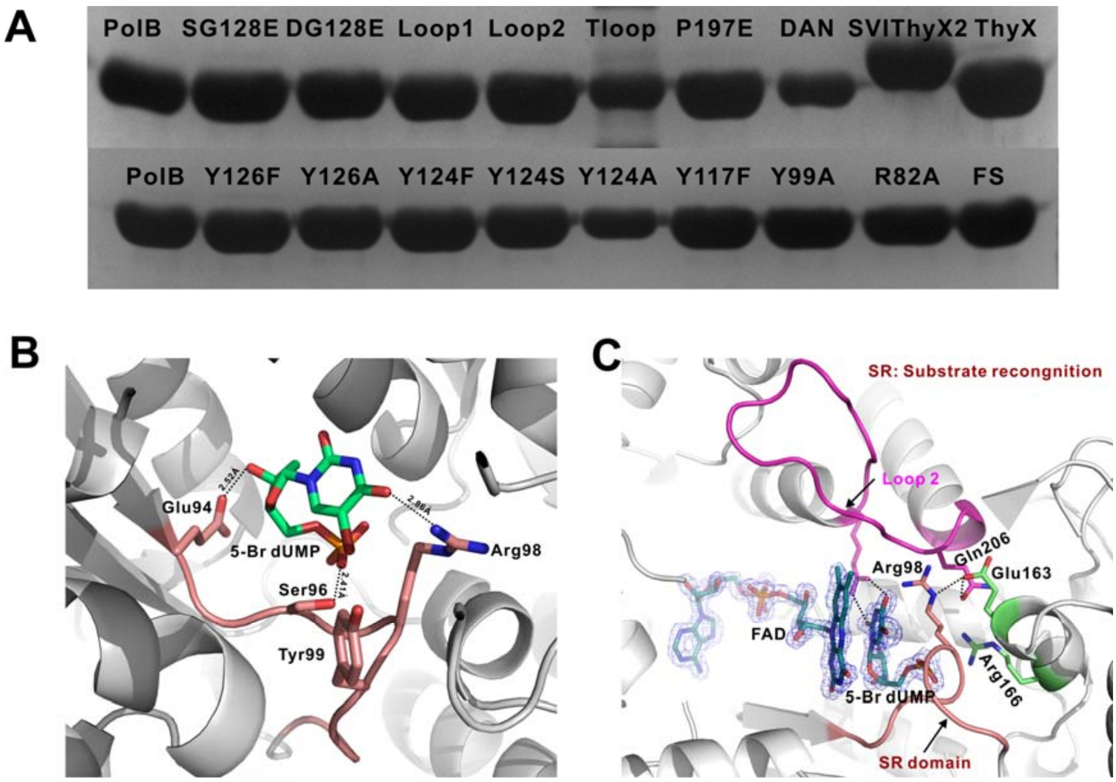

Figure S5. SDS-PAGE analysis of PolB variants, ThyX as well as SVIThyX2 (A) and The interactions of substrate and the substrate recognition peptide of PolB (B). (A) ThyX was from *S. cacaoi*, and SVIThyX2 was from *S. viridochromogens* DSM 40736. **(B)** and **(C)** All hydrogen bonds are indicated in dotted lines.

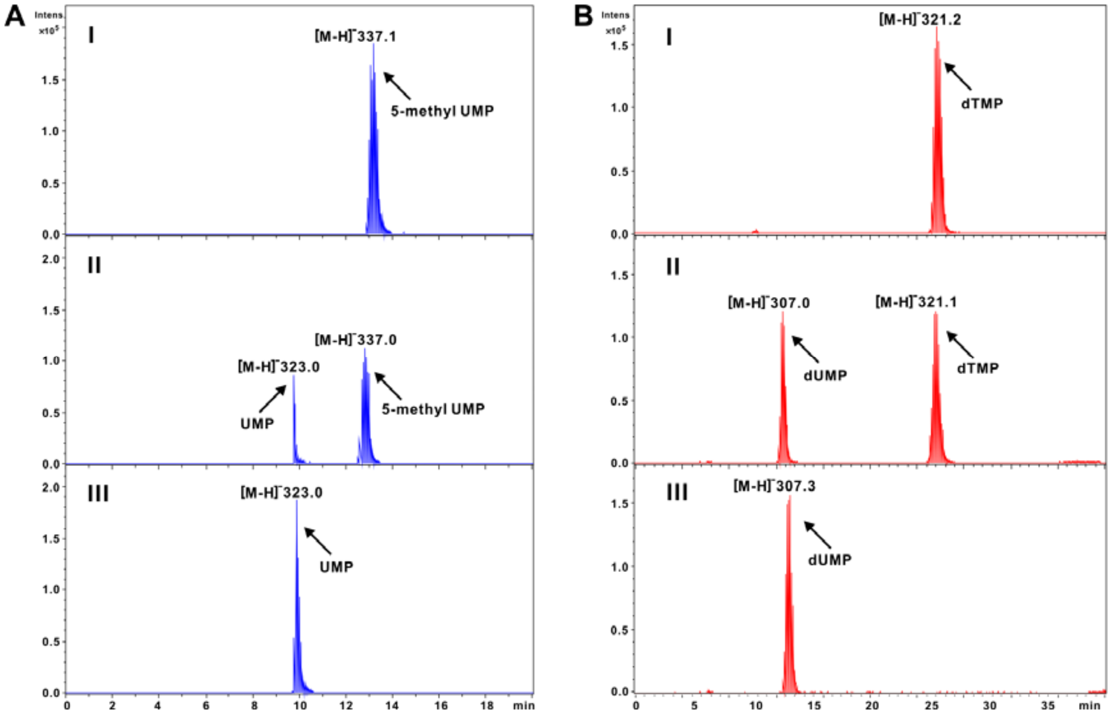

Figure S6. LC-MS analysis of the SVIThyX2 reaction using UMP and dUMP as substrates. (**A**) UMP as substrate. I, PolB with UMP; II, SVIThyX2 with UMP; III, UMP only. (**B**) dUMP as substrate. I, PolB with dUMP; II, SVIThyX2 with dUMP; III, dUMP only.

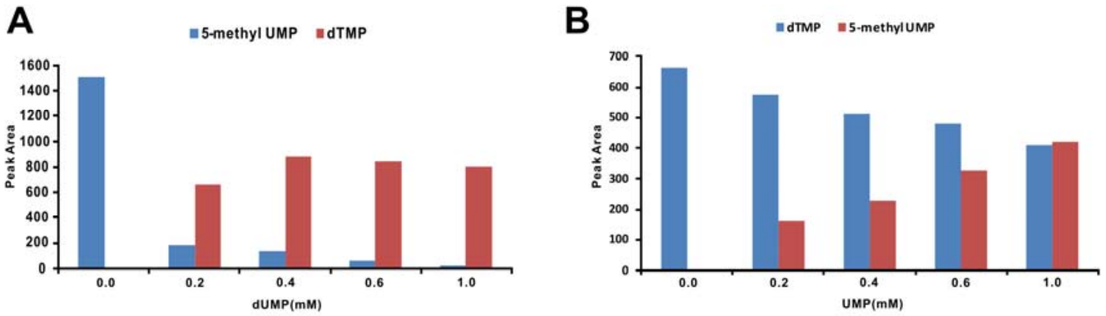

Figure S7. Competition assay for PolB using UMP/dUMP as substrate. (A) UMP (at fixed 0.2 mM) was competed by variable concerntration of dUMP (0, 0.2, 0.4, 0.6 and 1.0mM). **(B)** dUMP (at fixed 0.2 mM) was competed by variable concerntration of UMP (0, 0.2, 0.4, 0.6 and 1.0mM). The production of 5-methyl UMP and dTMP (peak area) were calculated for catalytic efficiency.

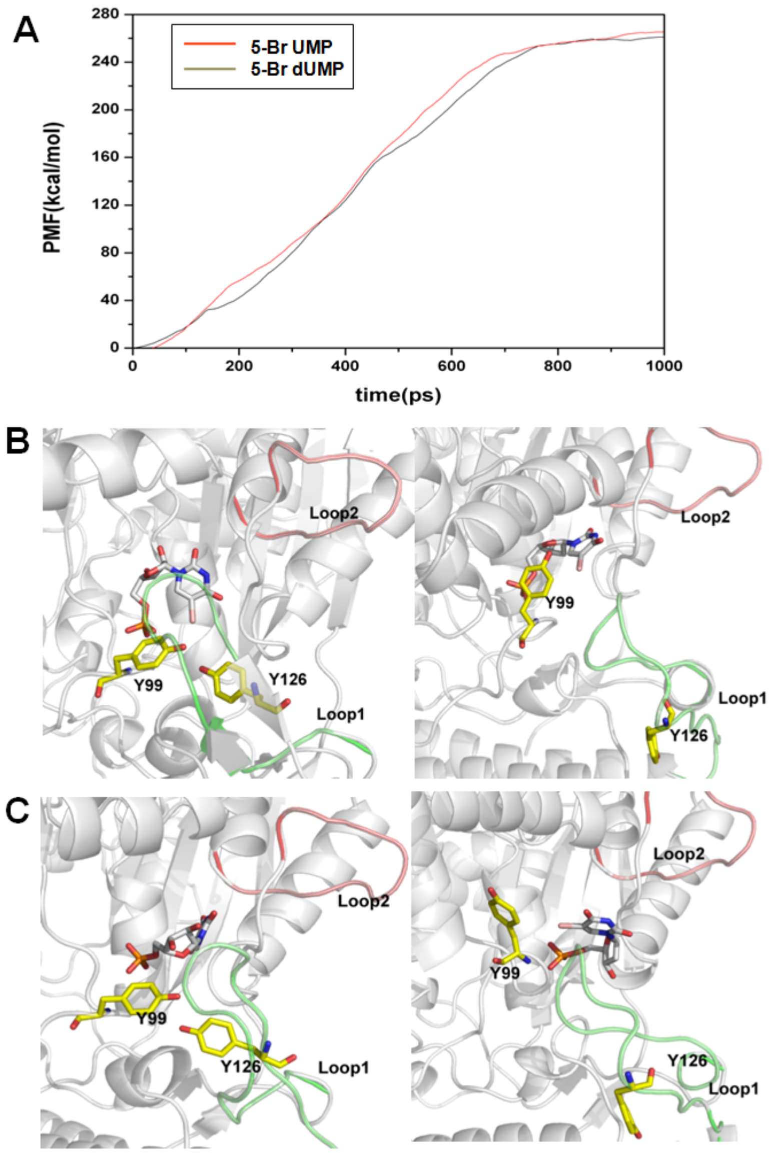

Figure S8. MD simulation results. (A) PMF profiles of 5-Br UMP (black) and 5-Br dUMP (red) dissociated from PolB protein. I, the fluctuation of ligand near the binding site; II, the movement of ligand in the channel formed by Loop1 and Loop2. **(B)** and **(C)** Conformations of residues Y99 and Y126 during the SMD simulations. **(B)**, left, PolB/5-Br UMP at 200ps; right, PolB/5-BrdUMP at 200ps; **(C)**, left, PolB/5-Br UMP at 500ps; right, PolB/5-Br dUMP at 500ps.

Reference

Chen, W., Huang, T., He, X., Meng, Q., You, D., Bai, L., Li, J., Wu, M., Li, R., Xie, Z.*, et al.* (2009).

Characterization of the polyoxin biosynthetic gene cluster from Streptomyces cacaoi and engineered production of polyoxin H. J Biol Chem 284, 10627-10638.
Kieser, T., M. J. Bibb, K. F. Chater, M. J. Butter, and D. A. Hopwood. (2000). Practical Streptomyces genetics. a laboratory manual. John Innes Foundation,. Norwich, United Kingdom.
